# Resistive expressions in preschool children during peripheral vein cannulation in hospitals: a qualitative explorative observational study

**DOI:** 10.1186/s12887-015-0508-3

**Published:** 2015-11-19

**Authors:** Edel Jannecke Svendsen, Anne Moen, Reidar Pedersen, Ida Torunn Bjørk

**Affiliations:** Department of Nursing Science, Institute of Health and Society, Faculty of Medicine, University of Oslo, Oslo, Norway; Centre for Medical Ethics, Institute of Health and Society, Faculty of Medicine, University of Oslo, Oslo, Norway

**Keywords:** Children, Exploratory methods, Pediatric, Relationships, Health care, Resistance, Restraint, Medical procedure

## Abstract

**Background:**

Children may resist common medical procedures, and this may lead to the use of restraint. This can be challenging to all of the involved parties, but empirical research is scarce on children’s expressions during these procedures.

**Methods:**

To explore preschool children’s resistive expressions during peripheral vein cannulation we video recorded and performed an in-depth analysis of naturally occurring situations with six newly hospitalized preschool children.

**Results:**

Fourteen attempts of peripheral vein cannulation were recorded. A typology of resistive expressions was developed consisting of: protest, escape, and endurance. During the expression of protest, the children showed an insistent attitude where they were maintaining their view. The expression of escape was when children were panicked, avoiding hands of adults when being approached. When expressing endurance the children were stiff, motionless and introverted. Less physical restraint is required during endurance, but children still appear to refuse participation.

**Conclusions:**

We identified three types of resistive expressions that can be used to better understand the individual child and inform clinical judgment in challenging procedural situations. This knowledge can help to sensitize health care providers in their attempt to arrange for children’s participation.

## Background

Hospitalized preschool children undergo many common but potentially painful and stressful medical procedures for diagnostic-and treatment-related purposes. Common procedures include peripheral vein cannulation (PVC), venipuncture, and nasogastric tube insertion. PVC is not an easy task in children and several attempts are often necessary to successfully place a PVC-needle. From a child’s perspective, PVC is a highly uncomfortable and uncommon event, and has been shown to create high levels of experienced pain, distress, and anxiety [1–3].

Several studies have reported methods of helping children through medical procedures. These studies suggest the need for local anesthetics such as lidocaine, and non-pharmacological approaches such as distraction, preparatory information, and the presence of parents [4–10]. It is important to focus on pain, distress, and anxiety in the care for children during procedures. However, this focus may contribute to an undesirable understanding of the children as passive or even irrational receivers of care, which in turn may hinder exploration of alternative interpretations and approaches to the situation [11, 12].

Physical restraint is often used to complete these common procedures [13, 14], and this might be harmful to the child [15], and challenging for the parents and the professionals. Restraint can be defined as use of force to overpower the child and is, by definition, applied without the child’s consent [16]. The importance of acknowledging the role of restraint was pointed out by Crellin [16]. Two studies have described preschool children’s resistance during immunization situations and found actions of rejection and reluctance towards the situation and their parents [17, 18]. A recent study explored children’s behaviour during the procedure of venipuncture [19]. The descriptions indicating resistance during the procedure were termed avoidance, forced engagement and resigned engagement.

Although children become increasingly competent in making rational judgments as they get older, refusal of treatment by a preschool child is a complex and multifaceted situation [20]. “Voice” in preschool children is typically more non-verbal than verbal [17], and younger preschool children may not have fully developed abilities to express feelings and opinions in nuanced words to show how they think. Therefore, data on their behavior could support interpretations on how they are affected. According to the Convention on the Rights of the Child, children have the right to participate in all situations that involve them [21]. Generally, young children’s perspectives in health care have not been sufficiently studied [11]. Observing preschool children’s expressions during procedures where restraining episodes can occur is important to better understand them [22].

### Symbolic interactionism

Perspectives of symbolic interactionism (SI) was chosen for this study because they can help to identify how people seek to understand the meaning of each other’s actions in a social interaction [23]. In line with SI one must try to understand how the children handle and interpret the meaning in the procedural situations [23]. Within symbolic interactionism one assumes that human beings act on the basis of the meanings that things have for them, and that these meanings are handled in and modified through an interpretive process used by the person dealing with the encounter [23]. Perspectives from symbolic interactionism were chosen because we were interested in the children’s meaning of the situation. A person’s use of meaning is seen as more than an application of their already established meanings. It is an interpretive process in which meanings are used and revised as instruments for the guidance and formation of action.

### Aim

This study aimed to explore children’s resistive expressions in situations of PVC, where they could be subjected to restraint. The following research questions were developed:

How do children express resistance when interacting with parents and health care providers?

How do children ascribe meaning to parent’s and health providers’ actions during the procedures?

## Methods

### Design

The present study is part of a larger qualitative study investigating a common medical procedure where restraint can occur. The study had an exploratory design because little is currently known about the phenomenon at hand [24]. A field study was designed, collecting observational and interview data and field notes from insertion of PVC. Because of anticipated difficulties in interviewing young children, interview data were collected from the nurses, physicians, and parents. In the present study data from video observations and field notes were included. Data from interviews and parent-health care provider interactions will be presented in later articles.

### Sampling, setting, and participants

The study was performed in a children’s medical unit situated in a large central teaching hospital in the southern part of Norway. The unit had approximately 20 beds and admits children who are 0–18 years old with non-surgical conditions, such as severe infections, cancer, and diabetes. A purposive, criterion sampling strategy was used to capture information-rich cases [24]. The inclusion criteria were that the child required PVC, was between 3 and 5 years of age, had less than three earlier admissions, the hospital stay to date was less than 14 days, and the child should not have an emergency condition. Because it was difficult to exclude children with experiences from earlier needle procedures, the goal was to avoid children who already had adjusted to a hospital stay with multiple medical procedures. The non-emergency condition allowed for time and the possibility for health care providers to make judgments about the use of restraint and alternative strategies.

Three girls and three boys, between 3 and 5 years, accompanied by their parents and other relatives participated in the study. Five of the children had infections and one was admitted because of stomach pain. Four of the children needed intravenous access for antibiotic treatment, one for rehydration and one for diagnostic radiology purposes. Only one child had an earlier hospital admission 2 years prior. All of the children were treated with local anesthetic cream on the expected skin area for cannulation. One child required twice medication with sedatives due to massive resistance to the procedure. The characteristics of the situations for each child are described in Table 1.

**Table 1:** Demographic and contextual characteristics of the patients

	Boy 1	Boy 2	Boy 3	Girl 1	Girl 2	Girl 3
Relatives involved	Mother	Mother	Father	Mother and other relative	Father	Mother and father
Nurses involved	1	1	1	3	1	1
Physicians involved	1^a^	1	1	2	1^a^	2
Child’s experience of procedures same admission	PVC and venipuncture	None	Venipuncture	PVC and venipuncture	Nasogastric tube and venipuncture	Venipuncture
Time hospitalized prior to PVC	5 days	12 h	1 day	1 day	1.5 days	3 h
Number of attempts to insert the PVC-needle	1^b^	1	2	4	3	3
Successful PVC	No	Yes	Yes	Yes	Yes	Yes

^a ^Same physician.^b^ In this situation, the PVC was aborted before perforation of the skin.

A total of seven physicians and nine nurses participated in the recorded situations. One of the physicians participated in two situations. All of the children had met at least one of the health care providers before the recorded incident. The physicians used a total of 14 attempts to successfully insert the cannula. One boy did not receive a PVC-needle (Table 1). All but one of the situations occurred in the unit’s treatment room. The remaining situation took place in the patient’s room because of a preliminary diagnosis of contagious stomach flu. Preparation of equipment by nurses prior to the procedure differed. In some cases, the nurse had been in the room to prepare for PVC before the family arrived. In other situations, the process to prepare the equipment started when the nurse came into the room together with the family.

### Data collection

Data were collected between May 2012 and May 2013 in six observed situations with a total of 14 attempts of PVC. The observed situations lasted between 10 and 94 min, starting 1–2 min before the participants entered the room, and lasting until the health care providers indicated that they were finished with the procedure. A video camera was placed on a tripod and the first author was present in the room during the procedure. To help participants forget the presence of the camera, the researcher positioned herself away from the camera. Field notes were written by the first author after each procedure. The video recordings enable the researchers to view the situations several times and to be analysed by the entire research team. By observing actions, we were able to discern what is taken for granted and discover what occurred in each situation [25]. Since preschool children have difficulties in providing detailed descriptions of their actions it is important to use methodologies that are sensitive for capturing their expressions and viewpoints [11].

### Ethics and protection of privacy

Approval from The Regional Research Ethics Committee South-East C (reference number 2011/2193), and the local research management in a hospital situated in the South-East Regional Health Authority was obtained. Data collection and storage were managed according to the laws and guidelines regulating research in Norway. Written informed consent was asked from health care providers and parents. The parent(s) also gave written consent on behalf of the child. No additional PVC was performed on a child for the purpose of this study.

### Analysis

We imported the field notes and the video recordings into NVivo10® (QSR International, USA), which is a soft ware solution made for managing and shaping unstructured qualitative data. The six situations involved 14 attempts to place the PVC-needle (Table 1). The children’s facial expressions, words and sentences, positioning, body movements, sounds and cries were described in detail using the built-in transcription tool of NVivo10®. This tool enabled parallel viewing and transcription.

The overall aim of the larger study was to explore the use of restraint during medical procedures. Reviewing the video recordings several times, we became aware of the children’s actions in the interaction and how resistance could represent the counterpart of restraint. The sensitizing concept “children’s resistance” provided a general sense of reference in approaching the empirical material. It enabled attention to variations in how the children displayed resistance during the different attempts [23]. An inductive content analysis was used [26] because it allows new insights to emerge from the data [27, 28]. The different descriptions of the children’s words and gestures were allocated to different NVivo10® -nodes. A node is a collection of references formulated according to the type and quality of data and could contain one or several similar descriptions. The next step of the content analysis was to cluster the nodes into the categories of expressions of resistance as shown in Table 2.

**Table 2:** Types of resistance expressed by children during PVC

	Expression of Protest	Expression of Escape	Expression of Endurance
Nodes on gestures	Presence of determined face with wide eyes and shut mouth Upright position on the parent’s lap Kicks and hits parents and health care providers or threaten to do so with the hand/foot Opposes attempts of comforting from parents Opposes removal of clothing, by holding on to them and pushing parents away Insistently avoids eye contact and look away on purpose Quickly looks at the health care provider’s face Answers questions and suggestions with “no” or “not” Cries for parents Short sentences not related to questions or examinations Argues for other needs Negotiates in a determined way Does not respond to reassurance Screams/shouts in an angry manner Increases volume of crying as a “warning” in response to adult verbal/non-verbal action	Fearful expression with wide eyes and open mouth Curls up and hides in the parent’s lap, constantly moving around on their lap. Points at other relatives in room Hides limbs in clothing Avoids comforting attempt from parents by moving the arm/body Uses the body to twist to avoid access to buttons and zippers Gaze seeks other adults outside the situation Gaze fixed on movement of adults Answers questions with “no/not” in a fearful manner Call for other activity Repeats call for parent although the parent is present Fearful voice when crying Does not respond to reassurance Cries or screams in a fearful, rapid manner Increases volume of fearful crying as a response to adult verbal/non-verbal action	Stone-faced or stiff facial muscles Body stiffness and distance from the parent’s body when sitting on the parent’s lap Ignores attempts of comfort from parents Body stiffness that hinders removal of textile Remote gaze, staring at point far away No answer or reactions to direct questions or examinations No follow-up on probes Ignores/does not hear commands Refuses to speak/ignores questions and suggestions No particular words or expression of sentences Does not respond to reassurance Cries in a monotonous continuous manner Increases volume of monotonous crying, but maintains the same pace of crying

In finalizing the analysis we highlighted the interactional aspects of the children’s expressions by using perspectives from SI. Within SI the term gesture is used to signify all verbal and non-verbal utterances. Interaction can be seen as a representation of gestures and a response to the meaning of those gestures [23]. The adult’s gesture is an indication or sign of what he is planning to do, as well as what he wants the child to do or to understand [23]. The child organizes his response on the basis of what the adult’s gesture means to him. These theoretical perspectives allowed for a deeper understanding of the children’s expressions and viewpoints.

## Results

Children resisted the PVC situations with different types of resistance: (a) protest, (b) escape, and (c) endurance. Resistance was the children’s way of showing their disapproval or disagreement. Children could display one or all of the types of resistance at different times of the procedure. Some of the children displayed the types of resistance in a weak manner, others in a stronger manner. To describe resistance, excerpts from situations with three children who resisted the procedures most strongly are presented below. These examples were selected because they contained the most condensed and illustrative information regarding how the children organized their responses on the basis of what the other participants’ gestures meant to them.

### Protest

Expressions of protest were observed when adults, either health care providers or parents, attempted to initiate contact, arrange for progress in the procedure, or attempted to touch the children. This expression was observed immediately after entering the procedure room, before the actual start of the PVC, and throughout different steps of the entire procedure. The interaction presented in Table 3 illustrates one example of protest. Boy 1 was supposed to obtain his second PVC during the hospital stay (Table 1). There had been one attempt to insert a PVC-needle earlier that day that had failed. Because there was no emergency the procedure was postponed until later. A new physician, who was unfamiliar with the family, was asked to do the PVC the second time.

**Table 3:** Excerpt from boy 1 regarding PVC

Participant	Actions (italics represent non-verbal actions)
Physician	May I have a look under there? Positive friendly voice (the physician is referring to a lidocaine pad with local anesthetics).
Boy 1	No-oh. The child looks determined with his gaze fixed on the physician’s hands while shaking his head.
Physician	No? Light voice and friendly tone.
Boy 1	The boy maintains a determined face and body position, and does not give an answer. He does not look at the physician. He keeps his hand in his sleeve.
Physician	Not at all? Keeps his voice light and has a friendly tone.
Boy 1	No answer. He still has a determined facial expression. He does not look at the physician.
Mother	Hmm?
Boy 1	He does not move. Determined facial expression maintained.
Physician	Can Rose^a^ look at your hand? Physician points at the nurse called Rose and smiles. Hmm?
Boy	The child shakes his head while looking down.

^a^pseudonym

The interaction demonstrates how the boy, using his facial and bodily expressions, turned down the physician’s invitation. The physician indicated what she was planning to do when she asked to get permission to inspect the hand, which was hidden within the boy’s sleeve. She further tried to obtain permission to remove the lidocaine pad with local anaesthetics. Body language and determination from the boy hindered progress of the procedure, despite the physician’s insistent, but friendly and positive approach. The child seemed to interpret the health care provider’s talk as bringing him closer to the insertion. The boy insistently ignored several attempts of contact by cutting off the conversation. The health care provider’s attempts to establish contact (and initiate the procedure) were met with a verbal protest of “no” and resolute facial expressions. At a later point in the procedure, he prevented his mother from removing his jacket by holding hard on to his sleeves from the inside and placing his hands over the zipper.

The expressions of protest took different forms in the children. The children appeared tense, sitting in an upright position on their parent’s lap. Some insistently avoided eye contact, and maintained a determined expression on their face, with the corners of their mouth pointing downwards and their chin down touching their chests. When looking at health care providers, they did so only for short periods of time, and looked away if the health care providers looked back at them. Crying, yelling, and screaming in a loud and angry manner were also characteristic for stronger expressions of protest, or repeating “no” or “not” or other short denial sentences. By repeating short sentences, shouting, and crying, the children drowned out the health care providers’ voices. They could also raise the intensity of their voice when they did not get any response to their protest, and when their protest was ignored for several times, their crying took form as “warning signals”. The most resistive children showed no actions that could indicate that they attached meaning to the adult’s suggestions or friendliness. However, the children, who displayed weaker signs of protest, cried and screamed less, and gave in more easily in to arguments from the adults. These children opposed the actions of the health care providers by not answering, thereby delaying progress. The children could also protest directly by refusing to follow direct commands or rejecting attempts of removing clothing by pushing the adults’ hands away.

### Escape

Expressions of escape were observed when adults, health providers, and parents, attempted to grab hold of them, or when the children realized that they were about to become overpowered. The interaction shown in the 12-second excerpt in Table 4 shows how girl 1 tried to escape during the first of four attempts to place a PVC-needle. Just before the excerpt starts, the health care providers tried to medicate her with a sedative to calm her down but, despite this, she was constantly screaming and moving back and forth on her mother’s lap. The mother attempted to hold her, while the health care providers tried to grab one of her legs.

**Table 4:** Excerpt from an attempt at sedation in girl 1

Participant	Actions (italics represent non-verbal actions)
Girl 1	No, no, no, no, no mummyyy. She screams the words out in a desperate way. Her eyes are focused on the health care provider’s arm. She displays a fearful expression on her face. Her body and legs are withdrawn from the adults who are attempting to grab hold of her feet while she is wriggling her legs.
Mother	The mother holds her child, preventing her from falling down from the bench. She has a tense look on her face.
Nurse	This is going just fine. The nurse adopts a positive tone while approaching the girl. She attempts to catch the girl’s wriggling leg in the air.
Girl 1	I don’t want a prick in my leg. Naaaeeeeeeeeeee. Screams loudly. Mummy, mummy, mummy, no, mummy. Screams louder and louder, and continues to wriggle her legs and flails her arms.
Relative	Mummy is holding.
Nurse	Hold the leg. The nurse points at the girl’s leg.

The excerpt shown in Table 4 demonstrates how the girl struggled to escape from the health care providers, by rapid movements and twisting of her body. The child had an alarmed facial expression and appeared to respond with immediate fear when her protest was ignored. She did not seem to catch the intended meaning of the positive tone and words of the health care providers. The kind words contrasted with the nurse’s struggle to take control. Instead, the girl watched their next movements, and attached meaning to their approaching hands. She raised the volume of her fearful cry, flailing and wriggling when the health care providers approached her.

Escape was variably expressed across situations and PVC attempts within situations. Escape was not observed without a prior protest, and now the child seemed to have modified his interpretation of the situation. Escape occurred when health care providers or parents decided not to listen to protests, but take direct actions. Consistently, during the expressions of escape, the children did not make eye contact with the parents or health care providers, and attached meaning more clearly to the health care provider’s movement. The children appeared alarmed and aroused on their parent’s lap, looking quickly around the room. They alternated their gaze between the health care providers’ bodily movements and a quick look around the entire room as if looking for escape. One child climbed onto her mother’s body to try to get away, while not letting the nurse’s hands out of sight at the same time. Crying and screaming in a fearful manner characterized escape. Repetition of sentences and words without pause and loud screaming were spontaneous expressions. This repetition appeared to be disconnected from the adults’ approaches. Without a break, the children shouted the name of the parents or called for help or release. One child screamed “ouch” repeatedly when the health care providers approached her and increased the tempo of “ouch” when the nurse looked at her, but still had not touched her arm. Some children screamed and shouted as if they were in severe pain and in a manner that affected their entire body when the adults threatened to or actually carried out their intentions. Another feature of escape was that it could be present in a short time interval. Escape often occurred when the health care providers stopped trying to make gestures of contact or to persuade the child, and decided to take physical action. The children displayed facial expressions of surprise and fright and fast body movements when they struggled to avoid the adults’ hands. They were startled just by the nurse passing by, e.g. when fetching equipment. Two of the children seemed to be incapable of powerful resistance or verbal protest because of their condition of illness. We zoomed in on their non-verbal expressions in the video recordings, and noticed that both of them hid their hands when the nurse or physician released their hand for some reason.

### Endurance

The children’s expressions of endurance comprised methods of self-restraint throughout the procedure. These expressions were observed during most steps of the procedure in some of the children, and in others at the end of an attempt where they had been through expressions of protest and escape. In the excerpt shown in Table 5, a girl gave no response when her father and the health care providers tried to talk to her. She sat stiffly, crying on her father’s lap, while the physician knelt on the floor below. The physician inspected her hand, and was concurrently attempting to communicate with her. Both of her hands were stiff and held out from her body. The inspected arm was lightly supported by the nurse.

**Table 5:** Excerpt of PVC in girl 2

Participant	Actions (italics represent non-verbal actions)
Girl 2	Nooo. The girl’s words are cried out in a monotonous way, staring into the air.
Father	He tries to drag his daughter closer toward him. This increases her body stiffness and her pitch of crying slightly rises.
Physician	Wow, did you make these? The physician points to the child’s bracelet, which is homemade of plastic pearls in different colors, and looks up into the child’s face and smiles.
Girl 2	I don’t want. Nooooo. The girl continues to cry in a monotonous way with a stiff body posture, and a stiff neck, and limbs. She sits in her father’s lap, ignoring the physician and fixes her glance on her arm where the physician holds her arm, not trying to withdraw the hand. Because of her stiff body, the father is unable to drag her closer to his stomach.
Physician	Or, maybe it is dad who has been sitting up and made it… ha ha ha ha (laughing) and looks first at the child, and then at the parent. Or what?
Girl 2	Noooooooo. The child still continuously cries in a rhythmic voice and is stiff in the body.
Father	He vaguely smiles and nods at the physician.

This excerpt demonstrates how the stiffened body posture and inflexibility in the child’s limbs communicates resistance in an introvert way. The girl did not respond to the health care provider’s intended meaning; neither to the humorous and inviting talk nor to the restraint. The stiffness of the girl made the adult’s efforts of contact and manipulation of her hand difficult and intrusive. The girl appeared to put energy into not moving, which also prevented her body from touching her father’s stomach, thus avoiding attempts at comfort. Her gaze appeared to be concentrated at something that was not present.

Expressions of endurance varied across situations and attempts at PVC. Words were expressed in a sore, rhythmic voice where they appeared to hinder interaction. Expressions of endurance comprised expressions of retreat and shielding from social interaction. The children appeared to prepare internally for something that was undesired. A tense and motionless body and facial stiffness were typical of endurance. The children did not actively avoid eye contact, but stared out into the air and did not respond to physical cuddling. During one attempt a child who was usually comforted by her pacifier showed no change in expression when this was removed or reintroduced. Endurance occurred during all attempts for one girl and only at some times for others. Those who had low energy went through the procedures with less stiffness, except during the actual needle prick. During endurance, the volume of the cry was moderate, and words were hardly used. The cries qualitatively changed in different ways according to the health care providers’ actions during the procedure. For example, when the needle prick was announced and inflicted or the tourniquet was tightened, the children intensified the rhythm and volume of the crying, but still focused on themselves. The children seemed to have stopped to attach meaning to the adults’ gestures. No actual reply to any direct question from adults was observed and the children displayed a suffering manner.

## Discussion

This study describes preschool children’s resistance to PVC procedures. The descriptions may contribute to nuance the existing accounts of children’s expressions of anxiety, pain, and distress because the focus is on how they organize their response on the basis of what the adults’ actions mean to them. The resistance consisted of expressions of protest, escape, and endurance. Each type of resistance involved distinct descriptions of gestures such as body posture, screaming, crying, or words and short sentences.

Protest was the most prominent type of resistance. Protest is recognized in many of Söderbäck’s [19] categories of engagement such as avoidance and forced engagement. However, the categories in Söderbäck’s study emanates from a different analytical perspective which makes a direct and detailed comparison difficult. One of Söderbäcks categories is forced engagement, however, our starting point was children who already were in risk of becoming, or were forced to be “engaged” in the procedure [19]. In the current study protest was identified during most steps of the procedure, also before the use of restraint. One interpretation of protest is that the children intended to hinder the health care providers in progressing with the procedure. A delay in progress was also identified by Harder et al. [17], who found that expressions of rejecting an invitation, turning attention away, taking their time, disapproving, and resisting, were part of 5-year-old children’s actions that delayed immunizations.

Protest seemed to escalate into escape when the children modified their interpretation of the health care provider’s actions. Gradually, they attached more meaning to the health care provider’s movements, and less to their talk. This can be understood as an interpretive process in which the children lost their belief in the adult’s talk as they realized that they were not being listened to, but ignored. The adults’ talk does not give meaning but their non-verbal actions guide the formation of the children’s actions [23].

During endurance the children seemed to “restrain themselves” by straining their muscles and directing their attention internally. Endurance seemed to mark a change in the children’s ascribed meaning of the situation, when they again modified their responses. During endurance, the children appeared to only interact with themselves, as similarly described by Söderbäck [19] in her study on venipuncture in children. Seemingly the belief in support from the adults had faded. To have lost trust in parents and health care providers in this situation may indicate a serious and lonely experience for the child that involves suffering [29]. In the current study, children required less (forceful) restraint during endurance than during other types of resistance. Crellin et al. differentiated the use of restraint in relation to how much force was used during medical procedures [16]. This indicates that the relationship between resistance and restraint is complicated, and that endurance needs further exploration to establish potentially harmful consequences for the child. The change in types of resistance throughout the procedure could be related to a lack of acknowledgment of the children’s views and feelings.

Changing between the different expressions, the children seemed to modify the meaning and what they attached meaning to in the situations. They actively attempted to make their opinion heard. This is similar to previous findings suggesting that pre-school children want to and do take an active part in health care situations [11, 12, 17–19]. They did not however, attach the meaning to the situation as the health care provider’s wanted them to. The children acted on the basis of the meanings that health care providers’ and parents’ gestures had for them [23]. For some preschool children who resist going through with procedures the adult’s gestures become unimportant. When children do not attach meaning to words, the use of interventions such as distractive talk seems less useful. Findings from several studies show that when children are forced, they often do not accept support, guidance or distraction [19, 30, 31]. 

Some of the children who displayed initial resistance continued to do so throughout the procedure. It seems that some children can keep on resisting and have difficulty in changing their course of action in terms of cooperation. Approaches used by health care providers and parents at the beginning of and during the procedure seemed to be ill-timed. Children’s low level of cooperation is a factor contributing to unsuccessful PVC [32] and often leads to more attempts to provide the child with an intravenous line, possibly resulting in an increasing number of restraining episodes. Therefore, children who initially resist a procedure may experience multiple attempts and multiple restraining episodes following the first procedure, something which requires special attention from health care providers. While the importance of children’s participation and consent is advocated [33–35] the present study confirms that participation and consent can be challenging for all the involved parties. To be able to achieve existing recommendations in clinical practice [21], the child’s views and feelings should be acknowledged. Even though it is not always possible to act in accordance with the child’s desires, it is still important to acknowledge the child’s perspective and competence [11, 36]. Findings from this study may enable health care professionals to identify various types of resistance in children, and to discuss and develop strategies for how to analyze, interpret, acknowledge, and deal with children’s resistance.

### Methodological issues

Although small samples are typical in qualitative research [24] we acknowledge that the findings were based on a small number of recorded situations. However, these recordings comprised 14 attempts that enabled a detailed study of the children’s expressions. Video recording with young children is a method which in a sensitive manner uncovers their expressions [11]. There are however limitations to the use of video recordings. Participants can change their behavior because of the camera and the presence of an observer. In this study, we explored children’s resistance, but we acknowledge that an important limitation is that we as researchers try to analyze the situations from the children’s perspective. We are only to some degree able to take their perspective [36].

One challenge of inductive content analysis is failing to develop a complete understanding of the context, which can result in findings that do not accurately represent the data [27]. To meet these challenges, 1 year was spent in the field indicating a prolonged engagement. The video recordings allowed for persistent observation in addition to data-and researcher triangulation. To increase the rigor of the interpretation, the researchers made independent interpretations of the data before discussing them together and compared expressions between children and across different attempts [37]. Although the sensitizing concept of resistance contributed to the prominence attributed to the stronger expressions of resistance during analysis, the concept may also have rendered us less sensitive to other phenomena and aspects of resistance. On the other hand the first author has had a professional role in a similar setting which can facilitate tolerance and sensitivity to such emotional situations.

## Conclusions

In this study we used perspectives from symbolic interactionism to interpret types of expressions in children’s resistance; protest, escape, and endurance. Protest was the most common type of resistance that was found during all phases of the procedure. Escape had a short timespan and was not identified without prior protest. Expressions of endurance indicated suffering and loneliness. Some of the children who displayed initial resistance did so throughout the procedure. The children seemed to modify the meaning and what they attached meaning to during the procedure, gradually detaching meaning from the adult’s gestures. The findings expand the former understanding of reactions which have mostly been addressed as pain, anxiety and distress. The descriptions of resistance might enable health care providers to elaborate on the child’s perspective and depict a child’s expression when consent and cooperation are challenging. Discrepancies between the child’s and the health care provider’s perspectives and feelings should be acknowledged and subject to reflections to enable the use of restraint with caution. If resistance to treatment is only understood as expressions of distress and pain, there is a risk that the child’s own perspective, opinion and other feelings might be neglected. Further research is required to investigate the usefulness of these concepts of resistance in clinical practice.

## Abbreviations

PVC: Peripheral vein cannulation
SI: Symbolic interactionism
